# A TMT-Based Proteomic Analysis of Osmoregulation in the Gills of *Oreochromis mossambicus* Exposed to Three Osmotic Stresses

**DOI:** 10.3390/ijms26062791

**Published:** 2025-03-20

**Authors:** Huanhuan Su, Dongmei Ma, Jiajia Fan, Zaixuan Zhong, Yuanyuan Tian, Huaping Zhu

**Affiliations:** 1 Key Laboratory of Tropical & Subtropical Fishery Resource Application & Cultivation, Ministry of Agriculture and Rural Affairs, Pearl River Fisheries Research Institute, Chinese Academy of Fishery Science, No. 1, Xingyu Road, Liwan District, Guangzhou 510380, China; hhsu2025@163.com (H.S.); madongmei2003@163.com (D.M.); fanjiajiaok@163.com (J.F.); zhongzaixuan0429@126.com (Z.Z.); tianyuan320@163.com (Y.T.); 2 Heyuan Branch, Guangdong Laboratory for Lingnan Modern Agriculture, Heyuan 517000, China; 3 Guangdong Provincial Key Laboratory of Aquatic Animal Immunology and Sustainable Aquaculture, Guangzhou 510380, China

**Keywords:** TMT-based proteomics, gills, osmotic stresses, *Oreochromis mossambicus*

## Abstract

Salinity and alkalinity are critical environmental factors that affect fish physiology and ability to survive. *Oreochromis mossambicus* is a euryhaline species that can endure a wide range of salinities and has the potential to serve as a valuable model animal for environmental science. In order to detect the histomorphological changes, antioxidant enzymes, and proteomic responses of *O. mossambicus* to different osmotic stresses, *O. mossambicus* was subjected to salinity stress (25 g/L, S_S), alkalinity stress (4 g/L, A_S), saline–alkalinity stress (salinity: 25 g/L, alkalinity: 4 g/L, SA_S), and freshwater (the control group; C_S). The histomorphological and antioxidant enzyme results indicated that salinity, alkalinity, and saline–alkalinity stresses have different degrees of damage and effects on the gills and liver of *O. mossambicus*. Compared with the control, 83, 187, and 177 differentially expressed proteins (DEPs) were identified in the salinity, alkalinity, and saline–alkalinity stresses, respectively. The obtained DEPs can be summarized into four categories: ion transport channels or proteins, energy synthesis and metabolism, immunity, and apoptosis. The KEGG enrichment results indicated that DNA replication and repair were significantly enriched in the salinity stress group. Lysosomes and oxidative phosphorylation were considerably enriched in the alkalinity stress group. Comparatively, the three most important enriched pathways in the saline–alkalinity stress group were Parkinson’s disease, Alzheimer’s disease, and Huntington’s disease. The findings of this investigation yield robust empirical evidence elucidating osmoregulatory mechanisms and adaptive biological responses in euryhaline teleost, thereby establishing a scientific foundation for the cultivation and genomic exploration of high-salinity-tolerant teleost species. This advancement facilitates the sustainable exploitation of saline–alkaline aquatic ecosystems while contributing to the optimization of piscicultural practices in hypersaline environments.

## 1. Introduction

Since fish spend all their lives in the water, several environmental conditions, including salinity, alkalinity, temperature, dissolved oxygen, and nitrogen content, have impacts on the development and survival of fish. Salinity and alkalinity are important factors in aquatic environments, which are primarily influenced by the geographical surroundings, geological soil, climates, and other factors [1]. Freshwater fish’s ability to endure salinity variations is directly threatened by the increases in sea level brought on by global warming and seawater inwelling brought on by tropical storms in estuaries and nearshore seas [2,3]. Extensive research has demonstrated that aquaculture animals under saline–alkalinity stress showed various adverse reactions, including slow growth performance [4], low feed utilization [5], impaired immune response [6], intestinal microbial composition [7], and nutritional metabolism disorders [8]. Salinity, which has long been acknowledged as one of the key environmental elements, affects the number of physiological processes in marine and estuarine animals, including survival, hemolymph osmolarity, and tissue water content [9,10]. Salinity adaptation is a complex process that necessitates the number of physiological responses to the environment, each with unique osmoregulation [11,12]. Additionally, the changing ion composition of high-alkalinity water, as well as the concentration and ratio of the major ions, is the primary factor restricting the life and growth of aquatic species [13,14]. Salinity and alkalinity may trigger the gills’ morphologic alterations since the gills are interacting directly with the surrounding water [15,16]. After being raised at salinities of 25‰, the hybrid tilapia (*Oreochromis mossambicus*♀ × *O. urolepis hornorum*) gill transcriptome responses were investigated [17]. A large number of genes related to osmotic stress regulation have been discovered and verified.

Indicators of antioxidants can, to some extent, show the adaptation effect of aquaculture animals that have acclimated to the salinity of the outside water [18,19]. According to several studies, alterations in environmental factors, such as water temperature and salinity, can affect the body’s ability to balance oxygen free radicals and cause oxidative pressure [20,21]. Oxidative damage truly happens when the body’s antioxidant system is unable to eliminate too many free radicals. The bulk of the body’s antioxidant defense system is made up of the enzymes catalase (CAT) and superoxide dismutase (SOD). SOD converts oxygen free radicals into hydrogen peroxide to protect cells from hydrogen peroxide toxicity and preserve the integrity of the body’s intracellular milieu. The hydrogen peroxide is then further broken down by CAT into oxygen molecules and water [22]. The body’s free radical metabolism may be properly assessed by variations in SOD and CAT activity, which is important for assessing the body’s capacity to combat free radicals and sustain health [23]. Glutathione peroxidase (GSH-PX), a vital peroxidase that may catalyze the conversion of glutathione to oxidized glutathione and the reduction of hazardous peroxides into non-toxic hydroxyl compounds, is essential for minimizing oxide interference with and harm to cell membrane structure and function [24]. When the body’s capacity for scavenging free radicals is overwhelmed by the body’s continual generation and accumulation of those radicals, oxidative pressure results [21]. Too many free radicals will harm the biofilm, causing lipid peroxides (LPOs) and, eventually, malondialdehyde (MDA) to develop [25]. MDA is a result of animal lipid peroxidation, and the concentration of MDA may be a good indicator of the degree of lipid peroxidation that has taken place. MDA is the byproduct of lipid peroxidation in animals, and its concentration can indicate the amount of lipid peroxidation, free radicals, and oxidative cell damage in tissues [26].

Tilapia is one of the most frequently farmed aquatic commercial species and a high-quality protein source for people around the globe due to its flexibility and adaptability to changing environmental conditions. The bulk of tilapia species are euryhaline, and *Oreochromis mossambicus* is unique in having an exceptional ability to osmotically adapt from freshwater to seawater, and more than 80 countries have introduced its species [27]. *O. mossambicus* may have a rather extensive salinity adaptation system, making it a good model for studying the molecular mechanisms regulating osmotic pressure [28]. By using this species as the study subject, investigating the osmotic pressure regulation mechanism can help scholars better understand the physiological and biochemical responses of other euryhaline fish to ambient salinity stimulation. However, studies on the osmotic stress regulation mechanism and the gill proteomics of *O. mossambicus*’ response to salinity, alkalinity, and saline–alkalinity are still lacking.

Tandem mass tags (TMTs) are widely used in quantitative proteomics to simultaneously identify and quantify proteins across diverse biological samples, enabling insights into molecular responses to environmental stressors [29]. Studies on aquatic species, such as salinity adaptation in fish gills [30] or cold tolerance in marine organisms [31], have leveraged TMT-based proteomics to reveal critical processes like energy metabolism, osmotic regulation, and stress response. These analyses highlight how environmental changes dynamically alter protein expression in tissues, offering a mechanistic understanding of adaptation. Proteomics thus serves as a vital tool for decoding molecular pathways underlying environmental resilience in aquatic systems.

In the current investigation, TMTs and LC-MS/MS were used for protein identification and quantification to examine the proteome of *O. mossambicus* in response to salinity, alkalinity, and saline–alkalinity stresses. These findings can help us comprehend how osmoregulation might work in aquatic species, particularly in euryhaline fishes. This work offers a comprehensive review of the gene expression patterns and pathways involved in osmoregulation in tilapia, which may help us better understand the molecular mechanisms under various osmotic stressors.

## 2. Results

### 2.1. Gill and Hepatic Histological

**Figure 1:**
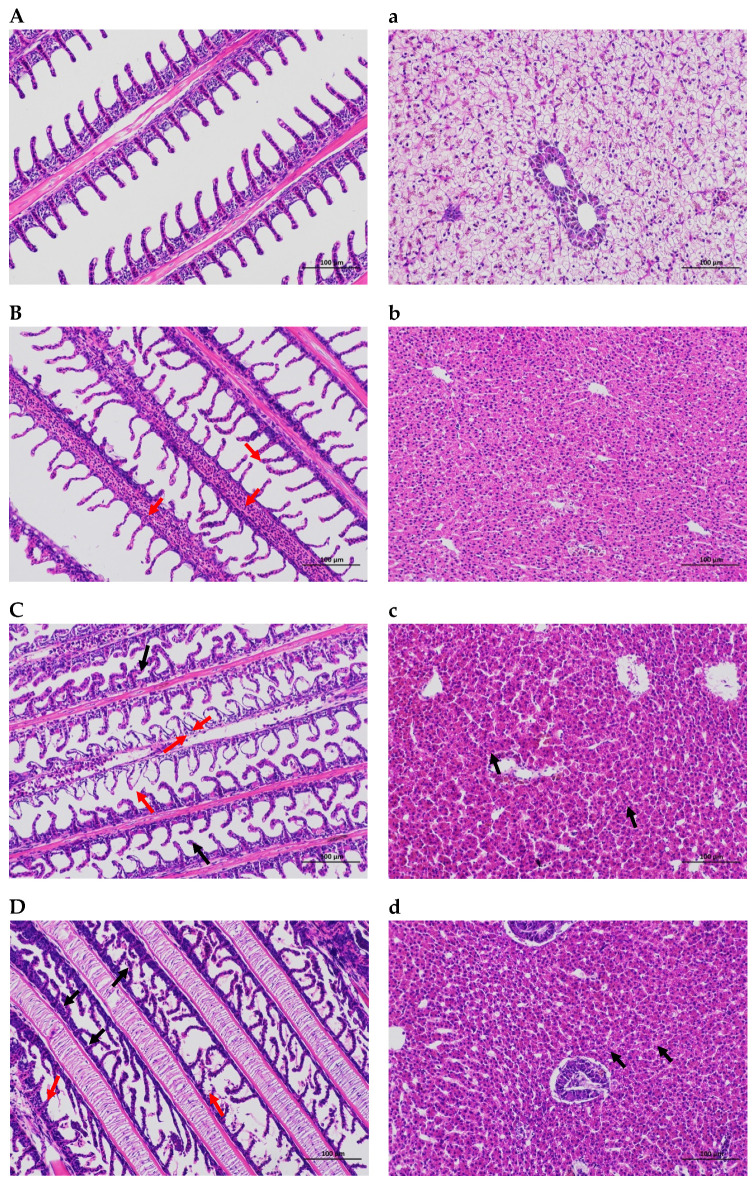
Histological analysis of the gills and livers of *O. mossambicus* under different osmotic stress. (HE stain, ×200) (**A**): the gills of the control group; (**B**): the gills of the salinity stress group; (**C**): the gills of the alkalinity stress group; (**D**): the gills of the saline–alkalinity stress group; (**a**): the livers of the control group; (**b**): the livers of the salinity stress group; (**c**): the livers of the alkalinity stress group; and (**d**): the livers of the saline–alkalinity stress group. Note: Black arrow: the gill flaps were arc-shaped; Red arrow: the lamellar epithelial cells separate from the basement membrane.

The microscopic morphology of the gills and livers of the control and stress groups was considerably different. The gill tissues of the three osmotic stress groups displayed inconsistent orientation of the gill flaps on the gill filament after exposure to salinity, alkalinity, and saline–alkalinity environmental conditions for 24 h. Many of the gill flaps were arc-shaped. Additionally, the group exposed to salinity stress had more blood vessels visible in the gill filaments and lamellae. In addition, a few lamellar epithelial cells were shown to separate from the basement membrane, and the gap widened in the alkalinity and saline–alkalinity stress groups. However, in the control group, the epithelial cells of the gill filaments were normal, the gill lamellae were arranged neatly on both sides of the gill filaments, the blood channels were clear, and the tissues were not abnormal. Compared with the control group, the liver tissues in the three stress groups showed mild loosening of the cytoplasm, occasional pigmentation, slight vascular congestion, diffuse fatty vacuolized hepatocytes, and no obvious necrosis or inflammation in the liver (Figure 1).

### 2.2. Biochemical Activity Analysis

**Figure 2:**
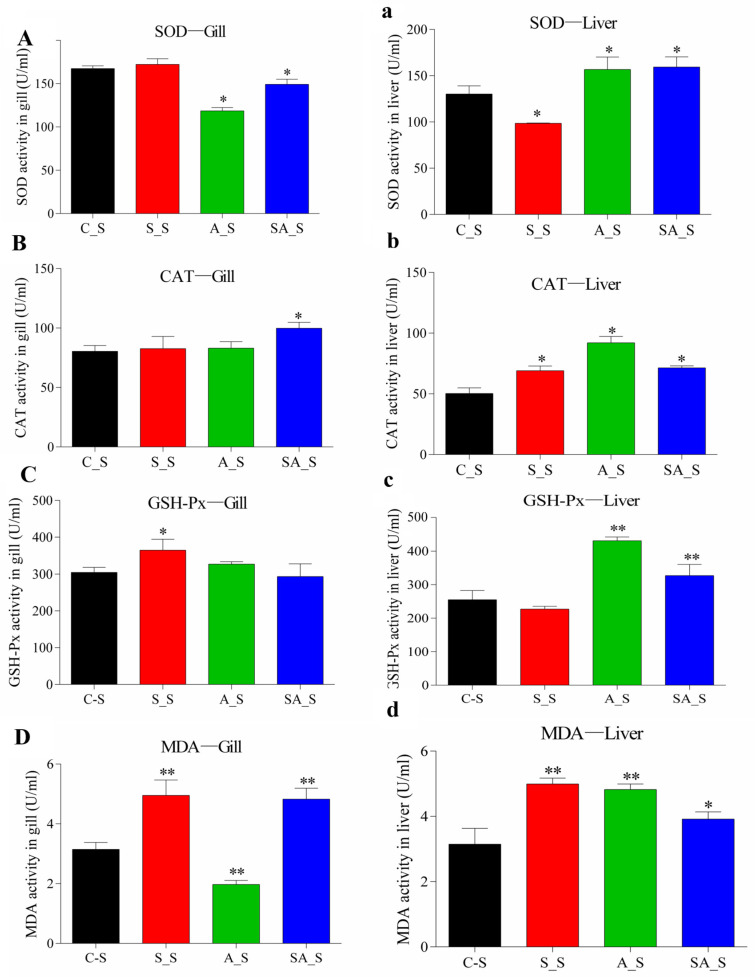
The activity of superoxide dismutase (SOD), catalase (CAT), glutathione peroxidase (GSH-PX) and malondialdehyde (MDA) in the gills and livers of *O. mossambicus* under three osmotic stresses. (**A**), superoxide dismutase (SOD) in gills; (**a**), superoxide dismutase (SOD) in liver; (**B**), catalase (CAT) in gills; (**b**), catalase (CAT) in liver. (**C**), glutathione peroxidase (GSH-PX) in gills; (**c**), glutathione peroxidase (GSH-PX) in liver; (**D**), malondialdehyde (MDA) in gills; (**d**), malondialdehyde (MDA) in liver. The different colored histograms represent different osmotic stresses, and the significant difference analysis only compares the same part of the control group. The asterisks above the histogram bars indicate significant differences (*: stands for *p* < 0.05; **: stand for *p* < 0.01) in the different stress groups in Duncan’s multiple range tests, n = 3. Note: C_S: the control group; S_S: the salinity stressed group; A_S: the alkalinity stressed group; SA_S: the saline–alkalinity stressed group.

Following a 24 h period of osmotic stress, the SOD activity in the alkalinity and saline–alkalinity groups in the gills was considerably lower than that of the control groups (*p* < 0.05); however, the activity of SOD in the liver was significantly higher in these two stress groups. The SOD activity in the gills did not change between the S-S groups and the control groups, but it was significantly reduced in the liver (*p* < 0.05). The activity of the CAT in the gills of *O. mossambicus* was considerably increased in the saline–alkalinity stress group (SA-S) compared to the control group (C-S) (*p* < 0.05) (Figure 2B). After being stressed for 24 h, the activity of the CAT levels in the hepatic tissue under salinity (S_S), alkalinity (A_S), and saline–alkalinity (SA-S) stress were considerably greater than in the control group (C_S) (*p* < 0.05). The activity of GSH-Px in the gill tissue of the salinity stress group was substantially higher than that of the control group compared with that of the control group (*p* < 0.05), while there was no significant difference in the alkalinity and saline–alkalinity stress groups. However, the activity of GSH-Px in the livers under alkalinity and saline–alkalinity stress was substantially greater than that in the control group (*p* < 0.05) (Figure 2C). Additionally, the concentration of MDA under alkali stress was substantially lower in the gill tissue than the control group (*p* < 0.05), while it was significantly greater in the liver tissues under the three osmotic stresses compared to the control group.

### 2.3. Proteomic Profiles in the Gills Under Osmotic Stress

The findings of the LC-MS/MS analysis yielded 388,715 total spectra, 48,486 spectra, 23,492 peptides, and 4730 identified proteins, of which 4715 proteins were quantified. An additional indication of the high quality of the proteomic data is that 43.21% of the sequence coverage distribution was higher than 10%. Numerous bioinformatics databases looked at the subcellular localization of the identified proteins. The nucleus included 25.97% (910 proteins) of the identified proteins, whereas the cytoplasm, extracellular space, and mitochondrion each contained 20.26% (710 proteins), 10.82% (379 proteins), and 10.73% (376 proteins), respectively.

#### 2.3.1. Proteomic Profiles in the Gills Under Salinity Stress

**Figure 3:**
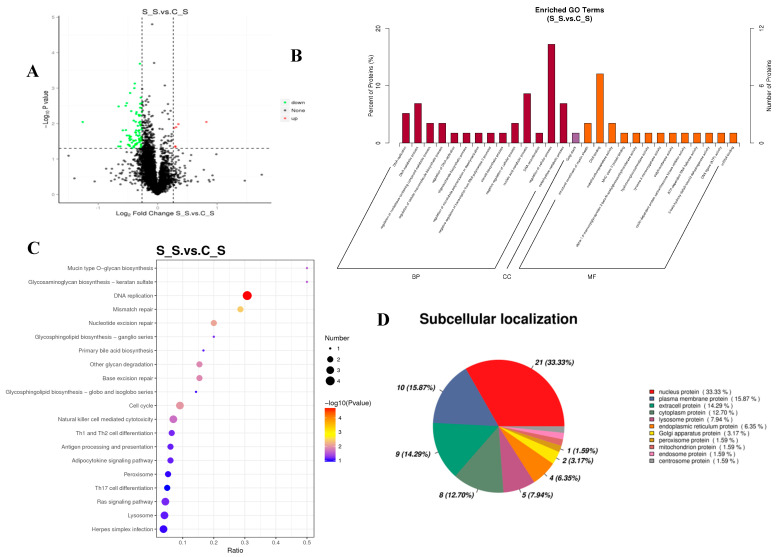
Function analysis of differentially expressed proteins S_S vs. C_S. (**A**): volcano plots of DEPs under salinity stress; (**B**): GO-based enrichment analysis of DEPs; (**C**): KEGG pathway enrichment analysis of DEPs; and (**D**): subcellular location of DEPs under salinity stress.

To understand the proteome responses of *O. mossambicus* under salinity stress, a proteomic analysis based on TMTs was conducted to detect the DEPs in the gills after 24 h of exposure. Based on the screen criteria of FC > 1.2 or FC < 0.83 and *p* < 0.05, a total of 83 differentially expressed proteins (DEPs) were found, including 6 differentially up-regulated proteins (DUPs) and 77 differentially down-regulated proteins (DDPs). The protein quantification statistics were shown on a volcano plot (Figure 3). The differentially expressed proteins for osmoregulation were found in this research, including aquaporin-3 (AQP3) and solute carrier family 12 member 2 (Slc12a2). A total of 28 substantially enriched GO terms were produced using the criterion of a corrected *p*-value < 0.05, and these terms were divided into three groups with 13, 1, and 14 terms for molecular functions, cellular components, and biological processes, respectively. Proteins were considerably enriched in the structural components of the myelin sheath (GO: 0019911), DNA binding (GO: 0003677), metallocarboxypeptidase activity (GO: 0004181), and other molecular functions for the 83 DEPs between the S_S and C_S comparisons. The Golgi stack (GO: 0005795) was considerably enriched for the cellular component group. DNA replication (GO: 0006260), DNA metabolism (GO: 0006259), control of nucleobase-containing chemical metabolism (GO: 0019219), and others were considerably enriched for the biological processes (Figure 3). Nine substantially altered KEGG pathways were found in the enrichment analysis of KEGG for the DEPs (*p*-value < 0.05). The top three enriched pathways among these were DNA replication (map03030), mismatch repair (map03430), and nucleotide excision repair (map03420) (Figure 3). Protein domain enrichment analysis was performed to show how the domains of proteins with varied expression levels changed. The up-regulated domain enrichment analysis revealed that seven pathways, including tyrosine metabolism (map00350), the prolactin signaling pathway (map04917), and others, were considerably enriched. Nine were highly enriched for the down-regulated domains, including DNA replication (map03030), mismatch repair (map03430), nucleotide excision repair (map03420), and others. Metabolic pathways, signaling, and biological activities are tightly tied to the locations of proteins within cells. The subcellular location of the proteins discovered in this work can shed light on their function and direct cellular-level research. In a eukaryotic cell, the cytoplasm is where most proteins are made. Newly created proteins are directed to the appropriate subcellular compartments, where they carry out their biological functions. A total of 63 DEPs were ultimately identified at 11 distinct subcellular sites in the current investigation. The nucleus (33.33%) and plasma membrane (15.87%) held the majority of the proteins, followed by extracellular (14.29%), cytoplasm (7.94%), and endoplasmic reticulum (6.35%) (Figure 3D).

#### 2.3.2. Proteomic Profiles in the Gills Under Alkalinity Stress

**Figure 4:**
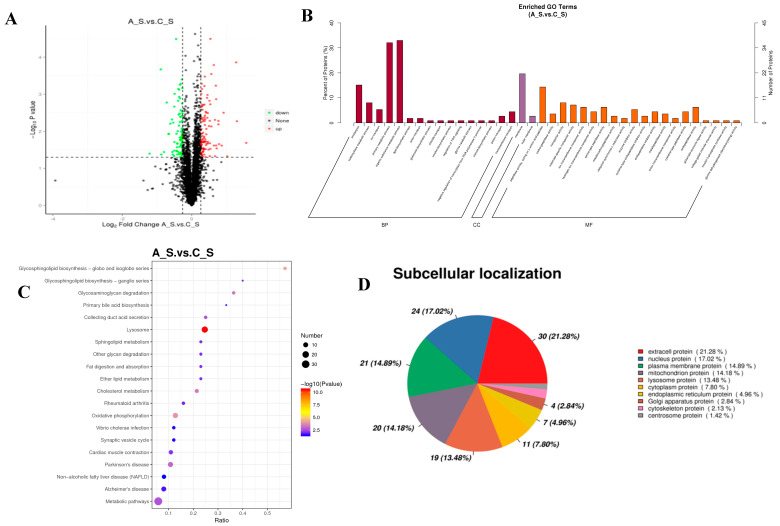
Function analysis of differentially expressed proteins in A_S vs. C_S. (**A**): volcano plots of DEPs under alkalinity stress; (**B**): GO-based enrichment analysis of DEPs; (**C**): KEGG pathway enrichment analysis of DEPs; and (**D**): subcellular location of DEPs under alkalinity stress.

The gill proteome was examined using TMT-based proteomics to better understand the proteomic responses brought on by alkalinity treatments. Alkalinity treatments caused substantial changes in 187 proteins, 107 of which were up-regulated and 80 of which were down-regulated (Figure 4). The information and descriptions of proteins with differential expression were found in this research. A total of 43 highly enriched GO keywords, divided into three groups with 25, 2, and 16 terms for molecular functions, cellular components, and biological processes, respectively, were found using the criterion of a corrected *p*-value < 0.05.

The proteins were considerably enriched in peptidase activity, operating on L-amino acid peptides (GO: 0070011), carboxypeptidase activity (GO: 0004180), transporter activity (GO: 0005215), and other molecular function activities for the DEPs between the A_S and C_S comparisons. The membrane (GO: 0016020) and outer membrane (GO: 0019867) were considerably enriched for the cellular component group. Proteolysis (GO: 0006508), carbohydrate metabolic process (GO: 0005975), ion transport (GO: 0006811), and other processes were highly enriched in the biological process (Figure 4). A total of 25 substantially altered KEGG pathways were found in the enrichment analysis of KEGG for the DEPs (*p*-value < 0.05). The top three enriched pathways among these were the lysosome (map04142), glycosphingolipid biosynthesis-globo and isoglobo series (map00603), and oxidative phosphorylation (map00190) (Figure 4).

Protein domain enrichment analysis was performed to show how the domains of proteins with varied expression levels changed. The down-regulated domain enrichment analysis revealed that a total of four pathways were highly enriched, including protein digestion and absorption (map04974), complement and coagulation cascades (map04610), type I diabetes mellitus (map04940), and ECM-receptor interaction (map04512). A total of 30 pathways, including the lysosome (map04142), oxidative phosphorylation (map00190), metabolic pathways (map01100), and others, were considerably enriched for the up-regulated domain enrichment. A total of 141 DEPs were ultimately identified at 11 distinct subcellular sites in the current investigation. The plasma membrane (14.89%), mitochondrion (14.18%), and lysosome (13.48%) were where the majority of the proteins were found, followed by extracellular (21.28%) and the nucleus (17.02%) (Figure 4D).

#### 2.3.3. Proteomic Profiles in the Gills Under Saline–Alkalinity Stress

**Figure 5:**
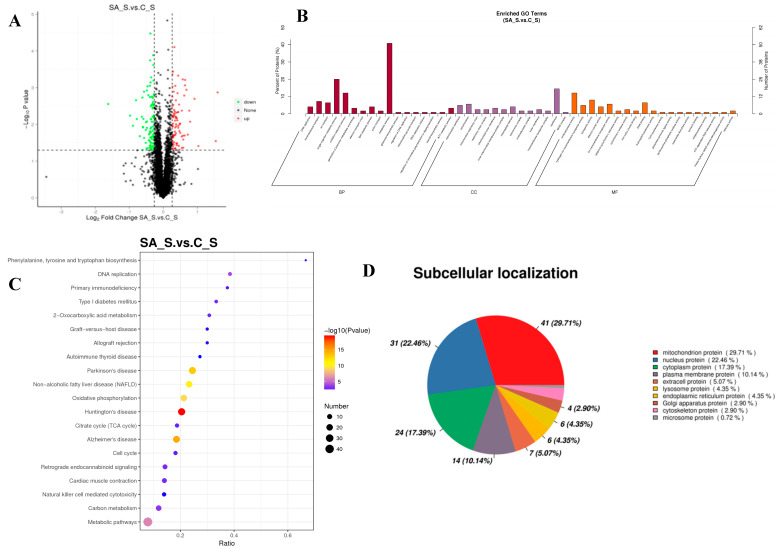
Function analysis of differentially expressed proteins in SA_S vs. C_S. (**A**): volcano plots of DEPs under saline–alkalinity stress; (**B**): GO-based enrichment analysis of DEPs; (**C**): KEGG pathway enrichment analysis of DEPs; and (**D**): subcellular location of DEPs under saline–alkalinity stress.

Utilizing TMT-based proteomics, the gill proteome was examined to learn more about the proteome responses of *O. mossambicus* during saline–alkalinity stress. Saline–alkalinity treatments caused substantial changes in 177 proteins, of which 92 proteins were down-regulated and 85 proteins were up-regulated (Figure 5). A total of 49 significantly enriched GO categories, divided into three groups, with 19, 13, and 17 terms for molecular functions, cellular components, and biological processes, respectively, were found using the criterion of a corrected *p*-value < 0.05. The proteins were considerably enriched in oxidoreductase activity (GO: 0016491), hydrogen ion transmembrane transporter activity (GO: 0015078), transporter activity (GO: 0005215), and other molecular function activities for the DEPs between the SA_S and C_S comparisons. The mitochondrial membrane (GO: 0031966), mitochondrion (GO: 0005739), mitochondrial respiratory chain (GO: 0005746), and others were highly enriched for the cellular component group. DNA replication (GO: 0006260), transmembrane transport (GO: 0055085), ion transport (GO: 0006811), and others were highly enriched in the biological process (Figure 5).

A total of 31 substantially altered KEGG pathways were found in the enrichment analysis of KEGG for the DEPs (*p*-value < 0.05). The top three significantly enriched pathways among these were Huntington’s disease (map05016), Alzheimer’s disease (map05010), and Parkinson’s disease (map05012) (Figure 5). The down-regulated domain enrichment analysis revealed a substantial enrichment of 19 pathways, including DNA replication (map03030), the NF-kappa B signaling pathway (map04064), the p53 signaling route (map04115), and others. A total of 28 pathways, including the cGMP-PKG signaling route (map04022), calcium signaling pathway (map040220), oxidative phosphorylation (map00190), carbon metabolism (map01200), citrate cycle (TCA cycle), and others, were substantially enriched for the up-regulated domains. A total of 138 DEPs were ultimately identified on 11 distinct subcellular sites in the current investigation. In addition to the nucleus (22.46%), the cytoplasm (17.39%), plasma (10.14%), and extracellular (5.07%) tissues held the majority of the proteins (29.71%) (Figure 5D).

### 2.4. Compared Proteomic Differences Between Osmotic Stresses

**Figure 6:**
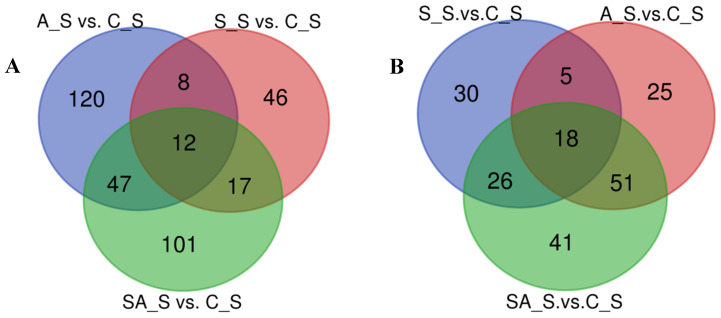
The DEPs number and Venn diagram of the overlap of the different stress groups. (**A**): the DEPs number and Venn diagram of the overlap of the S_S vs. C_S, A_S vs. C_S, and SA_S vs. C_S groups; (**B**): the KEGG pathways number and Venn diagram of the overlap of the S_S vs. C_S, A_S vs. C_S, and SA_S vs. C_S groups.

To further explore and elucidate the effects of different osmotic stresses on proteomic expression, 12 DEPs were found in all three treatment groups by comparative analysis of proteins in the gill tissues of *O. mossambicus* under three different osmotic stresses (Figure 6A). There were 8, 47, and 17 proteins expressed only in the A_S and S_S, A_S and SA_S, and S_S and SA_S groups, respectively. Additionally, there were 46, 120, and 101 significantly different expressed proteins that were uniquely expressed in the salinity stress, alkalinity stress, and saline–alkalinity stress, respectively (Figure 6A).

A comparison of the KEGG pathways that were enriched in the three groups revealed that 18 of the pathways, including metabolic pathways (map01100), cholesterol metabolism (map04979), arachidonic acid metabolism (map00590), fatty acid metabolism (map01212), and others, were enriched in all three groups (Figure 6B). However, only five pathways, including the osmoregulation-related pathway ECM-receptor interaction, were enriched in the salinity group and the alkalinity group (map04512). A total of 26 pathways were enriched in the salinity and saline–alkalinity groups, including the DNA damage repair pathways (base excision repair (map03410), nucleotide excision repair (map03420), and mismatch repair (map03430)) and pathways related to osmoregulation (MAPK signaling pathway (map04013), ras signaling pathway (map04014), and NF-kappa B signaling pathway (map04064)). A total of 51 pathways were enriched in the alkalinity stress group and the saline–alkalinity stress group, including those involved in amino acid metabolism and osmoregulation pathways, such as the pentose phosphate pathway (map00030), fatty acid elongation (map00062), oxidative phosphorylation (map00190), arginine biosynthesis (map00220), alanine, aspartate, and glutamate metabolism (map00250), glycine, serine and threonine metabolism (map00260), calcium signaling pathway (map04020), cGMP-PKG signaling pathway (map04022), p53 signaling pathway (map04115), mineral absorption (map04978), etc.

In order to elucidate the effects of the different osmotic pressures on *O. mossambicus* gills, 37, 7, and 18 differentially expressed proteins were enriched in the S_S vs. A_S, S_S vs. SA_S, and A_S vs. SA_S groups, respectively. Among the 37 significantly different proteins enriched in the salinity group compared with the alkalinity group, only one protein (Slc12a2) was up-regulated, and the rest were down-regulated. Compared with the saline–alkalinity stress group, all the differentially enriched proteins in the salinity group were down-regulated, while the 13 proteins in the alkalinity group were up-regulated and 5 were down-regulated. Between the salinity and alkalinity groups, 15 signaling pathways were significantly enriched, including lysosome (map04142), cholesterol metabolism (map04979), sphingolipid metabolism (map00600), pancreatic secretion (map04972), proximal tubule bicarbonate reclamation (map04964), etc. Compared with the saline–alkalinity stress group, the calcium signaling pathway (map04020), cGMP-PKG signaling pathway (map04022), olfactory transduction (map04740), vibrio cholerae infection (map05110), and salivary secretion (map04970) pathways were significantly enriched in the salinity stress group. Additionally, 22 pathways were significantly enriched in the A-S vs. SA-S group, including osmoregulation related pathways, such as proximal tubule bicarbonate reclamation (map04964), mineral absorption (map04978), thyroid hormone synthesis (map04918), endocrine and other factor-regulated calcium reabsorption (map04961), cAMP signaling pathway (map04024), cGMP-PKG signaling pathway (map04022), etc.

### 2.5. PRM Validation

**Figure 7:**
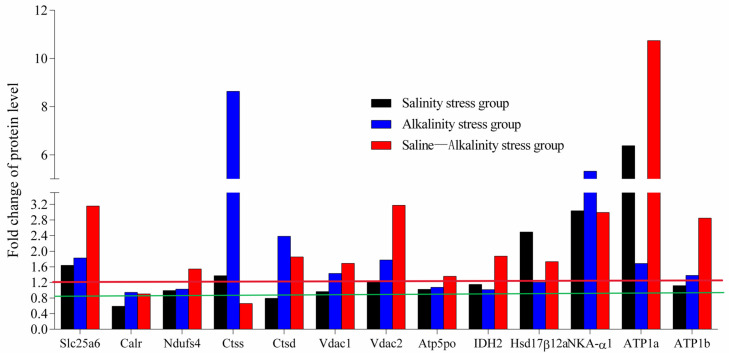
Relative quantification of the differentially expressed proteins in the gills of *O. mossambicus* under salinity, alkalinity, and saline–alkalinity stress using PRM analysis. The green line represents a fold change of 0.83, and the red line represents a fold change of 1.2. A fold change > 1.2 or <0.83 indicated that the protein was significantly differentially expressed.

A total of 13 proteins were validated in the three osmotic stress groups, and the PRM verification results showed that all of them were consistent with proteomics. Among these 13 proteins, Slc25a6, VDAC2, Hsd17β12α, NKA-α1, and ATP1α were up-regulated under the three osmotic stress groups, and VDAC1, ATP1β, CTSD were up-regulated in the alkalinity and saline–alkalinity stress groups, while CTSS was up-regulated in the salinity and saline–alkalinity stress groups. Additionally, ndufs4, idh2, and ATP5Po were up-regulated under saline–alkalinity stress, while calr was down-regulated under salinity stress (Figure 7). The changes in protein levels using PRM were, in general, consistent with those using TMT-based proteomic analysis results.

### 2.6. Osmoregulation Genes Expression

**Figure 8:**
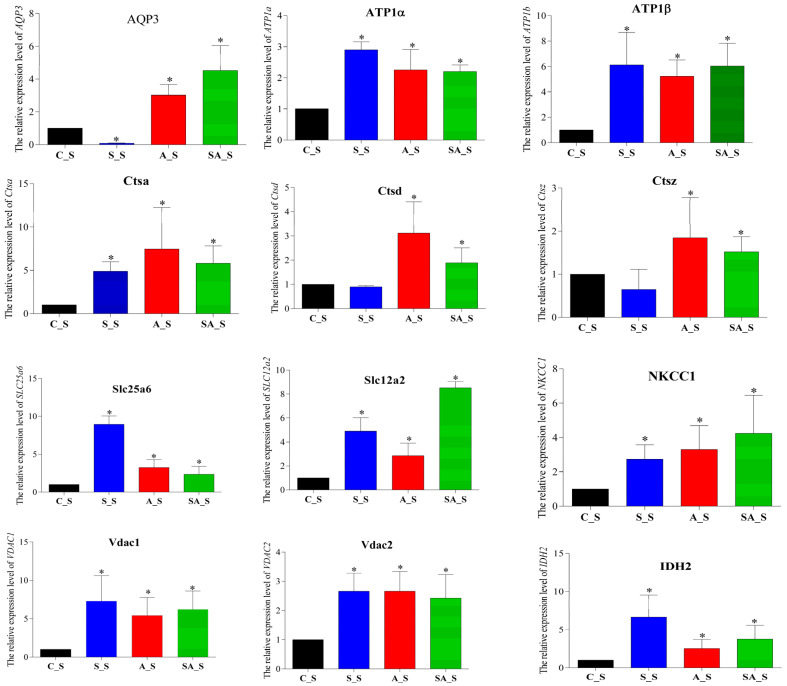
The expression levels of the significantly different genes in the control and the three osmotic stress groups. Note: the * represents that compared with the control group a significant difference level was reached (*p* < 0.05).

To assess the association between protein abundances and gene expressions and to further validate the findings of the TMT label-based quantitative analysis, qRT-PCR was carried out on a few chosen targets in both the osmotically stressed group and the control group. A total of 12 genes were examined, among which 9 genes’ mRNA expression levels were up-regulated under the three osmotic stresses (Figure 8). These results suggested that the change tendency of the gene expression levels was similar to those of the associated proteins.

### 2.7. Observation on the Apoptosis of Branchial Tissue Cells

**Figure 9:**
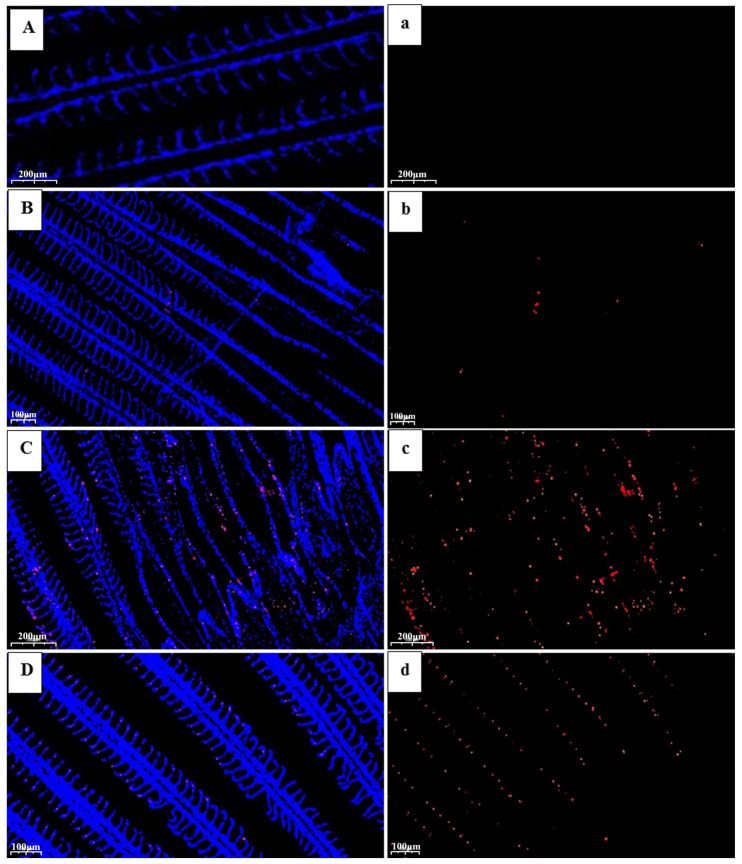
Apoptosis of the gill tissue cells of *O. mossambicus* in the control group (**A**,**a**), the salinity group (**B**,**b**), the alkalinity group (**C**,**c**), and the saline–alkalinity group (**D**,**d**). Note: the blue is normal cells and the red means the cells are apoptotic cells.

It was found that under salinity, alkalinity, and saline–alkalinity stress, Mozambique tilapia showed varying degrees of apoptosis (Figure 9). The apoptosis of the gill tissue cells under alkalinity stress was more serious than those under salinity and saline–alkalinity stress, indicating that alkalinity caused more serious damage to the gills of tilapia.

## 3. Discussions

The aquaculture environment is complex and dynamic, and a variety of water quality traits are connected to unpredictable farming conditions [32]. Freshwater fish species will be impacted by the deteriorating water quality caused by global warming [33,34]. Freshwater organisms cope with a variety of environmental conditions, including high salinity and alkalinity. Additionally, the challenges with survival and growth brought on by tissue damage and lower feed intake, as a result of erratic farming conditions, have significantly increased [35]. In the current study, *O. mossambicus* was subjected to salinity, alkalinity, and saline–alkalinity stress, and abnormal swimming and behaviors were shown after osmotic stress, showing a stress response to hyper-salinities and alkalinities.

### 3.1. Histologic Changes

It is generally recognized that gills play a significant role in respiration and are involved in a variety of physiological processes, including the excretion of metabolites, the regulation of body fluid permeability, and the control of acid–base balance, all of which are sensitive to osmotic stress. Therefore, the primary target organs that come into close contact with the surrounding water environment and osmolytes to cause toxic effects are the gills [36]. Meanwhile, gill health and pathological traits are often significant indicators for evaluating the aquatic environment [37]. In the present study, under salinity stress, the gills of *O. mossambicus* revealed slight congestion in the secondary lamellae, which was consistent with the report on Nile tilapia under salinity stress (salinity: 10‰) [38]. Additionally, by comparing the damage of gill tissue under the three kinds of osmotic stresses, it was found that alkalinity and saline–alkalinity caused more serious damage to the gill tissue of *O. mossambicus*, which further indicated that tilapia could tolerate higher salinity stress than alkalinity stress.

The liver, unlike the gills, is shielded from direct environmental exposure; nonetheless, because of its crucial function in active metabolism and detoxification, its reactions may also be noticeable [39]. Some research has revealed that the liver, the body’s primary metabolic organ, has been demonstrated to be a key supplier of carbohydrate metabolites for the osmoregulatory organs [40,41]. The liver tissue from *O. mossambicus* in this investigation had widespread fatty vacuolization and a mild vascular congestion, but no apparent necrosis or inflammation. These findings suggested that the liver responds to osmotic pressure more slowly than gill tissues.

### 3.2. Oxidative Stress Changes

Stressful environmental changes make it difficult for cells to operate properly and enable reactive oxygen free radicals (ROSs) to oxidize lipids, which causes lipid peroxidation [42]. In fact, excessive salinity has a variety of effects on aquatic species, starting with oxidative stress [43]. When cells are subjected to oxidative stress, SOD, CAT, and GPX are consequently activated to combat the elevated ROSs responsible for destroying DNA and impairing cell processes [44]. MDA levels are a marker of high lipid peroxidation levels and rise in response to stress [45,46].

Studies have shown that short-term high salinity can activate antioxidant enzymes, which can cause oxidative stress in tissues, including gills, intestines, and liver, which are essential for respiration, osmotic regulation, digestion, and detoxification [47,48]. In the current study, the liver of *O. mossambicus* subjected to alkalinity and saline–alkalinity stresses had higher activity levels of SOD, CAT, GPX, and MDA. However, the activity of SOD in the gills of *O. mossambicus* under alkalinity and saline–alkalinity decreased. Studies have reported that SOD activity was activated when teleost were subjected to mild stress, while under severe stress, SOD activity was inhibited [49,50]. The results of the present study indicated that alkalinity and saline–alkalinity caused more serious oxidative stress damage to the gills of *O. mossambicus* than salinity.

### 3.3. Gill Proteomics in Response to Osmotic Stresses

Compared to standard transcriptomics or genomics, which only evaluate messenger RNA, proteomics can be utilized to more directly define molecular reactions [40]. TMT-based proteome analysis has shown to be a potent method for locating and detecting DEPs among various samples under a variety of settings and stressors. It has been successful to use TMT-based proteomes to investigate how organisms adapt to various challenges in fish [51,52] and shrimp [53]. In the current work, the kinetics of protein expression in the gills of *O. mossambicus* under salinity, alkalinity, and saline–alkalinity stresses were examined. To the best of our knowledge, this is the first publication in which osmoregulation in *O. mossambicus* under diverse osmotic stressors was studied using TMT-based proteome analysis.

#### 3.3.1. DEPs Connected to Osmoregulation

Here, we could learn more about the proteomic reactions of the gills brought on under salinity stress by comparing the proteomes of the control and the stress groups. By analyzing the obtained significant differential proteins, it was found that these can be summarized into four categories: (a): ion transport channels or proteins (slc12a2, AQP3, VDAC2, etc.); (b): proteins related to the synthesis and metabolism of energy substances (idh2, atp6v1ba, atp6v0d1, etc.); (c): immune-related proteins (lcp1, myh10); and (d): apoptosis-related proteins (CTSA, CTSD, CTSZ, etc.).

Na^+^-K^+^-2Cl^−^ cotransporter 1 (NKCC1/Slc12a2) belongs to the SLC12A family of electrically neutral cationic chlorides and plays a role in maintaining electrolyte and fluid homeostasis [54]. NKCC1 is located in the basolateral membrane of osmoregulatory organs, helps regulate cell volume, and is highly expressed in ion-secreting epithelial cells [55]. Studies have shown that the expression of NKCC mRNA in gill tissues of European eels [56], *Fundulus heteroclitus* [57] and *Morone saxatilis* [58], increased after transfer to a seawater environment. The expression of NKCC1 is up-regulated in the gills of *Eriocheir sinensis* under salinity stress, suggesting that the expression level of NKCC1 can be increased under high salinity to accelerate the secretion of Cl^-^ in vivo, thus achieving salt and water retention [59]. Both acclimation and environmental ammonia exposure resulted in increased NKCC1 mRNA expression and protein abundance in the gills of *Anabas testudineus* [60]. It was also found that the expression of NKCC1 in *Fundulus heteroclitus* [61], *Petromyzon marinus* [62], and *Salmo trutta* [63] was significantly up-regulated after osmotic stress. These reports were consistent with the results of this study.

Aquaporin 3 (AQP3), a channel permeable to water, glycerol, urea, and ammonia/ammonium, can directly or indirectly participate in water transport, regulate cell volume changes through the transport of urea and glycerol, improve the osmotic sensitivity of ionic cells, and play a role in osmotic regulation [64]. Studies on *Dicentrarchus labrax* [65], *Anguilla anguilla* [56], Japanese eels [66], and Atlantic salmon showed that transferring fish from freshwater to saltwater drastically reduced AQP3 mRNA levels [67]. In this study, the expression level of AQP3 in the gill tissue of Mozambique tilapia under salinity stress was significantly lower than that of the control group. However, under alkalinity and saline–alkalinity stress, the expression level of AQP3 was increased, which was opposite to the salinity stress group. These results indicated that AQP3 plays a certain role in different osmotic pressure regulation. It may be that the function of AQP3 is activated under alkalinity and saline–alkalinity stress, as to carry out active transport of water outside the cell into the cell and maintain the balance of osmotic pressure inside and outside the cell.

NKA is a transmembrane protein mainly responsible for the active transport of Na^+^ and the entry of K^+^ into cells, which is not only important for the maintenance of cellular homeostasis, but also provides the driving force for many transport systems within the gills of fish [68]. It is also expected that the energy provided by ATP hydrolysis can be used to exchange intracellular Na^+^ for extracellular K^+^ [69]. Most euryhaline bony fish exhibit high NKA activity when living in seawater and are able to regulate the abundance of NKA in response to changes in salinity [27]. In the gills of bony fish, NKA is primarily located in specialized cell types called mitochondria-rich cells or ion cells, which are the sites of active ion transport. In this study, the expression levels of *ATP1α* and *ATP1β* in branchial tissue under the three osmotic stress groups were higher than those in the control group. The results of this study are consistent with those found in tilapia (*O. mossambicus*) [70], rainbow trout (*Oncorhynchus mykiss*) [71], *O. latipes* [72], *Salmo salar* [73], and the results of eel (*A. japonica*) [66].

Voltage-dependent anion channels (VDACs) were located in the outer membrane of mitochondria and form the boundary between mitochondria and metabolites. They act as gatekeepers for metabolites in and out and for mitochondria to communicate with other organelles. VDACs are also a key member of mitochondria-mediated apoptosis. VDACs are highly permeable to Ca^2+^ and regulate the entry of Ca^2+^ into the endometrial space to participate in mitochondrial permeability changes. Keinan et al. [74] found that Ca^2+^ binding sites exist on VDACs, and when VDACs are open, Ca^2+^ flows into mitochondria via VDACs. The result is mitochondrial swelling, mitochondrial permeability transition pore opening, and apoptosis. In this study, it was found that VDAC1 and VDAC2 were significantly up-regulated under salinity, alkalinity, and saline–alkalinity stress, and both were significantly higher than the control group. These results indicated that VDAC1 and VDAC2 had regulatory functions in response to the osmotic stresses.

Cathepsin is a lysosomal cysteine protease that exists in animal tissue cells, especially in lysosomes, and is mainly involved in mitochondria-mediated apoptosis under pathological conditions. According to the different amino acid residues in the active site, it can be roughly divided into three categories: cysteine protease, aspartic protease, and serine protease [75]. In this study, Cathepsin A (CTSA), CTSD, and CTSZ were expressed in the gill tissue of *O. mossambicus*, which was consistent with the results of studies on turbot (*Scophthalmus maximus* L.) [76] and *Pagrus major* [77]. In addition, it was found that a large number of cell apoptosis occurred in the gill tissue of *O. mossambicus* under alkalinity stress, which was more serious than those in the salinity and the saline–alkalinity stress groups, indicating that alkalinity caused more serious damage to the gill tissue of *O. mossambicus*. In the embryonic development stage of *Sparus aurata* and grass carp, the expression of CTSD was on the rise, and it was believed that CTSD was involved in body development by mediating programmed cell death [78]. CTSZ, a lysosomal cysteine protease of the papain superfamily, is involved in immune defense through phagocytosis, proliferation, and migration of immune cells. In *Cyprinus carpio*, it is widely expressed in tissues and plays a key role in yolk metabolism [79].

#### 3.3.2. Osmoregulation-Related Pathways

Through KEGG enrichment analysis of differential proteins, a total of 79, 99, and 136 pathways were enriched in the salinity, alkalinity, and saline–alkalinity stress groups, respectively, among which 9 pathways, 25 pathways, and 31 pathways had significant differences. In the salinity stress group, the significantly different pathways were mainly related to DNA replication and repair, and some osmoregulation pathways were also found in the gills of tilapia under salinity stress, such as the prolactin signaling pathway [80], the PPAR signaling pathway [17], and the MAPK signaling pathway [81], which have been reported in several aquatic animals. Osmoregulation-related pathways, including oxidative phosphorylation, the mTOR signaling pathway, ECM-receptor interaction [82], cell adhesion molecules (CAMs) [83], proximal tubule bicarbonate reclamation [84], etc., were enriched in the gill proteomic of *O. mossambicus* under alkalinity stress. Additionally, in the saline–alkalinity stress group, the calcium signaling pathway [85], proximal tubule bicarbonate reclamation, mineral absorption, ABC transporters, fatty acid metabolism, peroxisome, MAPK signaling pathway, etc., were also enriched in the gill proteomic of *O. mossambicus*.

**Figure 10:**
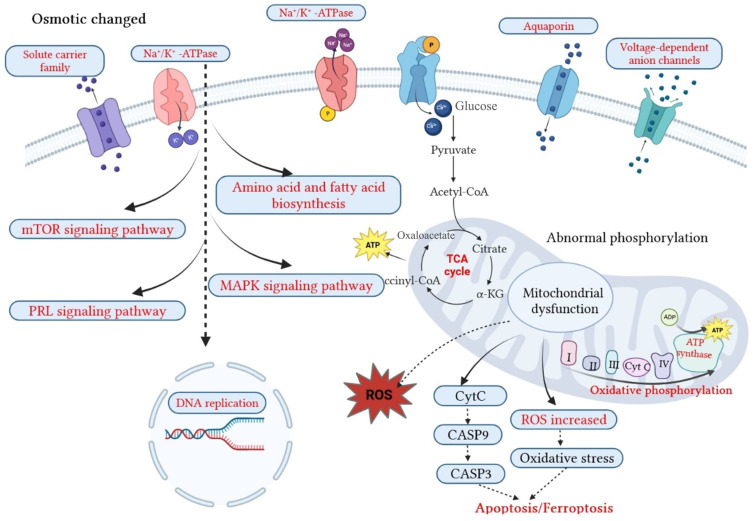
Proposed signaling pathways for three osmotic stresses in gills of *O. mossambicus*. Enriched pathways were labeled in red, and black ones were unchanged or not detected in current work.

The osmotic regulation networks in the gills of Mozambique tilapia under osmotic stresses were mapped by analyzing the pathways obtained under the three osmotic stresses (Figure 10). Under the stimulation of external osmotic pressure, ion channels or ion transporters located on the cell membrane are activated first, such as the aquaporin family (AQP3), the solute transport family (Slc12a2, Slc25a6), and NKA (ATP1α and ATP1β). After that, it induces the activation of relevant signal transduction pathways in cells, such as the mTOR signaling pathway and the MAPK signaling pathway, and activates DNA replication and related repair pathways. At the same time, it can stimulate the pathways related to energy synthesis in the cell, including the biosynthesis and metabolism of amino acids, the biosynthesis of long-chain polyunsaturated fatty acids, oxidative phosphorylation, citric acid cycle, etc. The stimulation of external osmotic pressure will cause certain oxidative damage to the fish body. When the body cannot remove the oxygen free radicals in the body, the expression of genes related to apoptosis will be activated, resulting in apoptosis.

Mitogen-activated protein kinases (MAPKs) have been shown to have crucial roles in the regulation of intracellular metabolism, gene expression, and important activities in a number of situations, including growth and development, disease, apoptosis, and cellular responses to external stimuli [86]. It has been discovered that MAPKs play a crucial role in the response to osmotic, thermal, and oxygen stresses [87]. Osmosensor signals can be sent via MAPKs to activate the necessary targets and regulate physiological acclimatization [88]. Through enhanced phosphorylation in response to osmotic stress, MAPKs may possibly change the apoptotic process and boost cellular survival capability [89]. These results indicate that MAPKs play a significant role in salinity adaptation. Our findings support the MAPK pathway’s involvement in the osmoregulation of *O. mossambicus*.

In this study, pathways including energy-related ones like glycolysis/gluconeogenesis, the citrate cycle, and oxidative phosphorylation that are critical for osmoregulation and the response to salinity change, were emphasized. The inner membrane of the mitochondria in eukaryotic cells is where oxidative phosphorylation occurs, which produces the majority of the energy for cellular functions [90]. Of the five complexes involved in oxidative phosphorylation, Complex I, succinate dehydrogenase (Complex II), Complex III, Complex IV, and ATP synthase make up the majority (Complex V). In this study, both alkalinity and saline–alkalinity treatments increased the expression of the majority of the members of the complex of oxidative phosphorylation. It is noteworthy that more DEPs were produced by the alkalinity and saline–alkalinity stressed groups than by the salinity stressed group, indicating that these groups’ effects on boosting electron transport and the contribution of molecules to oxidative phosphorylation were more severe. In response to saline–alkalinity stress, the up-regulated Complex II was connected to the up-regulated TCA cycle. In aerobic organisms, the TCA cycle serves as the primary metabolic route and the center of the metabolism for amino acids, lipids, and carbohydrates. Isocitrate dehydrogenase, pyruvate dehydrogenase, and succinate dehydrogenase, which were all up-regulated in the gills of *O. mossambicus*, were among the DEPs linked to the TCA cycle that were found in this study in the saline–alkalinity stress group.

Complex II and III are the primary locations for ROSs creation in the electron transport chain [91,92]. Complex III was up-regulated under saline–alkalinity stress, suggesting that there was an excess of ROSs produced. The electron transfer from reduced cytochrome c to molecular oxygen is carried out by Complex IV, and Complex IV is up-regulated in response to saline–alkalinity stress, indicating increased oxygen demand. The last enzyme in the process, Complex V, produces ATP from ADP by using proton gradients across a membrane [93]. Complex V was up-regulated in response to saline–alkalinity stress, showing that osmotic stress boosted ATP generation in *O. mossambicus* gills. Additionally, mitochondrial dysfunction and substrate mobilization may be to blame for the energy loss. Similar results were found in the hepatopancreas of juvenile olive flounders (*Paralichthys olivaceus*) [5] and freshwater crabs (*Sinopotamon henanense*) [94] that had been exposed to cadmium. Together, our results show that saline–alkalinity treatments increased the electron flow into the gill mitochondria, indicating increased energy needs in *O. mossambicus*’ gills.

An important defensive mechanism against stressors is apoptosis, a controlled and unique kind of planned cell death that eliminates surplus, old, necrotic, damaged, or possibly dangerous cells [95,96]. In this proteome investigation, cathepsin L was shown to be down-regulated in the gills of *O. mossambicus* during saline–alkalinity stress. This protein has recently been shown to trigger the death of midgut cells by activating caspase-1 during insect midgut remodeling [97].

Osmoregulation requires additional energy consumption [11]. Through hemolymph osmoregulation and ionic regulation, euryhaline invertebrates may adjust to changes in the ambient salinity [98]. Numerous enzymes and transporters participate in iono- and osmoregulatory processes, and the synthesis and function of these transport-related proteins consume a lot of energy [99]. Ye et al. [98] also found that the shrimp must increase their metabolic rate, which is linked to the osmotic pressure reaction and drives the regulating process, in order to adapt to the salinity shift. Although the exact amount of energy needed for ion- and osmoregulation is uncertain, aquatic species certainly expend some energy to manage their internal osmotic concentration [100]. Oxygen intake and ammonia excretion increase when aquatic species are subjected to decreasing salinity, and osmoregulation is greatly increased when there is an adequate supply of energy [101,102]. Our study identified the citrate cycle (TCA cycle), oxidative phosphorylation, and glycolysis/gluconeogenesis as the enriched energy metabolism-related pathways. The iono- and osmoregulatory mechanisms appear to be given the energy they need to adapt to shifting ambient salinity and alkalinity as a result of this consequence. Additional energy from these pathways is used to activate the synthesis and activities of Na^+^-K^+^-ATPase and other transporters or enzymes in the gills, intestines, and other osmoregulatory organs under osmotic stress, especially under alkalinity and saline–alkalinity stress conditions. This is in addition to the requirement for other metabolic processes that are involved in the response to salinity challenges [103,104].

## 4. Methods and Materials

### 4.1. Animals, Stress Exposure, and Sample Collection

*O. mossambicus* were provided by the Chinese Academy of Fishery Sciences Pearl River Fisheries Research Institute (Guangzhou, China). Prior to the stress exposure experiment, healthy fish were acclimated in a large outdoor net cage (pH 7.8, temperature 26–29 °C). The fish were given commercial feed twice a day (09:00, 17:00) at a rate of 5% of their body weight.

All healthy fish were randomly assigned to four groups after two weeks of acclimatization: the control group (C_S: fresh water), the salinity stress group (S_S), the alkalinity stress group (A_S), and the saline–alkalinity stress group (SA_S). Three duplicates of each treatment were included, and 100 fish (average weight of 28.75 ± 2.32 g) were housed in each tank (140 L, 74 cm × 53.5 cm × 41.5 cm). The salinity and alkalinity of the three treatments were set as the control (C_S: fresh water), the salinity stress (S_S: 25 g/L), the alkalinity stress (A_S: 4 g/L), and the saline–alkalinity (SA_S: salinity: 25 g/L and alkalinity: 4 g/L), respectively. The salinity, alkalinity, and saline–alkalinity stress concentrations for this investigation were set at 25 g/L, 4 g/L, 25 g/L, and 4 g/L, respectively, based on previous studies [105,106]. The control group was kept in fresh water during the whole experiment. Up until the ultimate concentration was attained, the salinity and alkalinity concentrations of the stress groups were started at a salinity of 10 g/L and an alkalinity of 1 g/L and raised by 5 g/L and 1 g/L every two days, respectively. The experiment was started after achieving the desired concentration. All treatments were aerated during the whole experiment to maintain consistent oxygen saturation levels. During the experiment, the water temperature varied from 28 to 30 °C, and the dissolved oxygen changed from 8.50 mg/L to 9.53 mg/L. During the whole experiment, the pH value under salinity stress was 8.2–8.5, the pH value under saline–alkalinity condition was 8.7–9.0, and the pH value under alkalinity stress was 9.3–9.5. After the experiment, the concentration of ammonia nitrogen in the aquarium was 0.01–1.65 mg/L, and the concentration of nitrite was 0.03–0.09 mg/L.

The captured fish were anesthetized with MS-222 (100 mg/L) [27] and dissected on ice after 24 h of exposure. The gills and livers of *O. mossambicus* were obtained for antioxidant enzyme activity and histomorphological analysis. The gills were also obtained and frozen in liquid nitrogen for proteomic analysis. Three fish were pooled into each biological duplicate, with three replicates in each group. Prior to experimental analysis, all samples for biochemical activity analysis and proteomic analysis were immediately frozen in liquid nitrogen. The gills and livers were fixed in buffered paraformaldehyde (4%) for histomorphology observation.

### 4.2. Histomorphological Observation and Antioxidant Enzyme Analysis

The obtained gills and livers were fixed with paraformaldehyde and made transparent with xylene. Then, samples were dehydrated by gradient anhydrous ethanol and embedded in paraffin. The samples were divided into sections with a thickness of 5 μm using a Leica RM2235 microtome (Leica Microsystems, Wetzlar, Germany). The tissue slices were conventionally stained with hematoxylin and eosin and were captured on camera utilizing the Olympus DP25 (Olympus Corporation, Tokyo, Japan) automated microscopy system with an Olympus BX-51 microscope.

Using commercial kits, the activities of the enzymes superoxide dismutase (SOD), catalase (CAT), glutathione peroxidase (GSH-PX), and the lipid peroxidation product malondialdehyde (MDA) were assessed in the gills and livers of *O. mossambicus* (KeyGen Biotech, Nanjing, China) following the manufacturer’s directions.

### 4.3. Proteomic Analysis

#### 4.3.1. Proteins Extraction and Digestion

The gill tissue samples were ground into powder at a low temperature and quickly transferred to a centrifuge tube, pre-cooled by liquid nitrogen. An appropriate amount of protein cracking solution (100 mM ammonium bicarbonate, 8 mM urea, pH = 8) was added, then shaken and mixed, followed by ultrasonic treatment in an ice water bath for 5 min to fully crack. After centrifugation at 4 °C for 15 min at 12,000× *g*, the supernatant was taken at 56 °C, and 10 mM DTT was added for 1 h, and sufficient iodoacetamide was added for 1 h at room temperature and away from light. At −20 °C, four times the volume of pre-cooled acetone was added and precipitated for at least 2 h. After centrifugation at 12,000× *g* at 4 °C for 15 min, precipitation was collected. An amount of 1 mL of pre-cooled acetone was added to the precipitation at −20 °C, and the precipitation was rinsed. The obtained precipitates were centrifuged at 12,000× *g* at 4 °C for 15 min. The collected precipitates were air-dried, and then an appropriate protein solution was added to dissolve the precipitated proteins. The standard curve with the absorbance of the standard protein solution was drawn and the protein concentration of the sample was calculated.

#### 4.3.2. TMT Labeling and Separation of Fractions

Protein samples were taken and added to the protein solution (8 M urea, 100 mM TEAB, pH = 8.5) to make the volume reach 100 μL. Pancreatic enzyme and 100 mM TEAB buffer were added to the mixture for enzymatic hydrolysis at 37 °C. Formic acid was added to bring the pH below 3 and was mixed well. The solution was centrifuged at 12,000× *g* at room temperature for 5 min, and the supernatant was slowly removed through the C18 desalting column. Then, the supernatant was cleaned continuously 3 times with the cleaning solution (0.1% formic acid, 3% acetonitrile), and an appropriate amount of eluent (0.1% formic acid, 70% acetonitrile) was added. The filtrate was collected and lyophilized. An amount of 100 μL of 0.1 M TEAB buffer was added for resolution, and 41 μL of acetonitrile-dissolved TMT labeling reagent was added, and the reaction was reversed at room temperature for 2 h. Then, the reaction was terminated by adding the final concentration of 8% ammonia. Samples marked with equal volume were mixed, and then lyophilized, after salt removal.

Liquid A (2% acetonitrile, 98% water, with ammonia adjusted to pH 10), and liquid B (98% acetonitrile, 2% water) were prepared as mobile phases. The freeze-dried powder was dissolved in liquid A and centrifuged at 12,000× *g* at room temperature for 10 min. The determination was performed on a Waters BEH C18 (4.6 × 250 mm, 5 μm) column with the column temperature set at 45 °C, using an L-3000 HPLC system (Novogene, Beijing, China). One tube was collected per minute, combined into 10 fractions, and dissolved with 0.1% formic acid after freezing.

#### 4.3.3. High-Performance Liquid Chromatography Analysis

The mobile phases, liquid A (100% water, 0.1% formic acid), and liquid B (80% acetonitrile, 0.1% formic acid), were prepared. The EASY-nLC TM 1200NA (Thermo Fisher/LC140, Shanghai, China) upgraded UHPLC system, Q-ExactiveHF-X mass spectrometer (Thermo Fisher, Shanghai, China), and NanosprayFlex (ESI) (Novogene, Beijing, China) ion source were used. The ion spray voltage was set at 23 kV, the temperature of the ion transmission tube was set at 320 °C, and the mass spectrum adopted a data-dependent acquisition mode. The full scanning range of MS was *m*/*z* 350–1500, the resolution of primary MS was set to 60,000 (200 *m*/*z*), the maximum capacity of the C-trap was 3 × 10^6^, and the maximum injection time of the C-trap was 20 ms. The parent ions with the top 40 ionic intensities in the full scan were selected to be splintered by the high-energy collision cracking (HCD) method, and the secondary mass spectrometry detection was carried out. The secondary mass spectrometry resolution was set at 45,000 (200 *m*/*z*), the maximum C-trap capacity was 5 × 10^4^ and the maximum C-trap injection time was 86 ms. The peptide fragmentation and collision energy were set at 32%, the threshold intensity was set at 1.2 × 10 s, and the dynamic discharge resistance range was set at 20 s. The original mass spectrometry data (.raw) was generated.

#### 4.3.4. Identification and Quantification of Proteins

The protein database was searched separately for the spectrogram of each peptide using the database search software Proteome Discoverer 2.2 (PD2.2, Thermo Fisher, Shanghai, China). The database search parameters were set as follows: The mass tolerance of precursor ions was 10 ppm, and that of fragment ions was 0.02 Da. The immobilized modification was cysteine alkylation modification, the variable modification was methionine oxidation and TMT labeling modification, and the n-terminal modification was acetylation and TMT labeling modification, allowing up to 2 missing cut sites. In order to improve the quality of the analysis results, the PD2.2 software further filtered the search results. Peptide-Spectrum Matches (PSMs) with a confidence level of more than 99% were identified as trusted PSMs. Proteins containing at least one unique peptide segment were identified as trusted PSMs. Only trusted peptides and proteins were reserved. FDR verification was performed to remove peptides and proteins with an FDR greater than 1%. The *t*-test was used for statistical analysis of the protein quantitative results, and the protein with a significant quantitative difference between the experimental group and the control group (*p* < 0.05 and FC > 1.2 or FC < 0.83, [fold change, FC]) was defined as a differentially expressed protein (DEP) [107,108].

#### 4.3.5. Functional Analysis of Proteins and DEPs

InterProScan software [109] was used to annotate GO and IPR functions (including Pfam, PRINTS, ProDom, SMART, ProSite, and PANTHER databases). COG and KEGG analyze the functional protein family and pathway of the identified proteins. Volcanic map analysis, cluster heat map analysis, and GO, IPR, and KEGG pathway enrichment analysis were conducted for DPE [110], and the STRING DB software (V9.1, http://STRING.embl.de/) was used to predict the possible protein–protein interactions (http://STRING.embl.de/). A two-tailed Fisher’s exact test was used to examine the enrichment of the differentially expressed proteins versus all identified proteins for GO annotation, KEGG enrichment, and InterPro database analysis. Using conventional false discovery rate control techniques, multiple hypothesis testing was adjusted, and domains with a corrected *p*-value < 0.05 were deemed significant.

### 4.4. Verification of Protein Identification by Parallel Reaction Monitoring (PRM)

To accomplish relative or absolute quantification of target proteins or peptide segments, targeted proteomics techniques, known as PRM (parallel reaction monitoring), were used. PRM is based on high-resolution and high-accuracy mass spectrometry. Five further DEPs were quantified using LC-PRMS analysis to confirm the differential abundance of proteins discovered using TMT-based proteomic analysis. First-stage mass spectrometry on a quadrupole mass analyzer (Q1) with high resolution and high precision (like the Orbitrap series) was used to preferentially capture the target peptide precursor ion. Finally, all of the target peptide’s ion information was gathered. On the nLC-1200 simple system, tryptic peptides were fed onto the stage tips of C18 for desalting before reversed-phase chromatography (Thermo Scientific). For PRM analysis, the Q Exactive Plus MS was utilized. The raw data was analyzed using Skyline (MacCoss Lab, University of Washington), and signal intensities for particular peptide sequences for each of the significantly altered proteins were evaluated with respect to each sample and normalized to a common reference.

### 4.5. qPCR Analysis of Key Gene Expressions

The gills of *O. mossambicus* were prepared for total RNA extraction, RNA integrity analysis, cDNA synthesis, and qRT-PCR as previously reported, after being exposed to osmotic stressors for 24 h [17]. Based on the impacts shown in proteomics and osmoregulation, eleven genes were chosen to be validated by qPCR. Using “Primer-Blast” (http://www.ncbi.nlm.nih.gov/tools/primer-blast/), primers (Table 1) were created, their concentration was tuned, and the results were verified using 1.5% gel electrophoresis. PowerUp^TM^SYBR^TM^ Green Master Mix fluorescence quantitative PCR reagent was used in the ABI StepOne Plus fluorescence quantitative PCR instrument, and *β*-actin was used as the internal reference gene. Using the 2^−ΔΔCt^ technique, the relative change in gene expression was determined [106].

### 4.6. Tunel Staining

The gill tissues of the control group, the salinity group, the alkalinity group, and the saline–alkalinity group were fixed, eluted with gradient alcohol, embedded, and sliced. The derivatives formed by deoxyribonucleotide and luciferin were labeled to the 3’-terminal of DNA for the detection of apoptotic cells, and observation and photography were performed.

### 4.7. Statistical Analysis

The means and standard deviation for three replications are reported for every data point. In this study, analysis of variance (ANOVA) was first performed to detect significant differences between the means of the different groups, and then Tukey’s test was applied for pairwise comparison. Using the SPSS 21.0 software to compare the significant differences between the treatments and the control group (SPSS Inc., Chicago, IL, USA), a *p*-value < 0.05 was regarded as significant.

## 5. Conclusions

In the present study, histological, biochemical, TMT proteomic, LC-MS/MS, and PRM were used to analyze the effect of osmotic stress on the gills of *O. mossambicus* and its underlying molecular regulatory mechanisms. The live performance, histological, and biochemical activity results indicated that alkalinity and saline–alkalinity caused more serious tissue injury and oxidative stress damage to the gills of *O. mossambicus* than salinity. This observed phenomenon could potentially be attributed to the pH in the water environment being higher under the saline–alkalinity and alkalinity stressed groups. The potential osmoregulation network was explored using KEGG analysis, and the ion transport related pathways, the pathways related to synthesis and metabolism of energy, and the apoptosis-related pathways were found to be the major significant enriched pathways. This study provides basic data for the study of the molecular regulation mechanism of osmotic pressure in tilapia and provides a theoretical basis for the expansion of tilapia culture area and the development and utilization of saline–alkali water resources.

## Figures and Tables

**Table 1:** Fluorescent quantitative PCR primers for functional genes.

Gene	GenBank Cod	Forward Primer Sequence (5′-3′)	Reverse Primer Sequence (5′-3′)	Length (bp)	Annealing Tm °C
*Slc25a6*	XM_003445230.5	GCGGGTAACCTGGCATCTGG	ATCAGCGGCGAGACGTGTTC	93	60
*IDH2*	DQ465384.1	ACAGACTTCCTCGACGCCAT	AGTGACATTGCCATCTTCGTGCT	94	60
*ATP1α*	XM_025903006.1	ACTGTCATGGGCCGTATCGC	CCAGGAAGACGGCCACTCCA	115	60
*ATP1β*	XM_003454723.5	TCTGGCTTGGAGGACACCGA	GATCACGTTGGGCTGCACCT	153	60
*AQP3*	XM_013264600.3	CCATTCCTTGGCGCCATTCT	CCTGCTTCTTGTCACGTGCT	88	60
*Slc12a2*	XM_003444449.5	GAGCCCGACAGTCCCTCTGA	CATCGCTCTGCGGTCGTGAT	232	60
*CTSZ*	XM_003441502.5	GCTCATGCCACGGAGGAGAC	GTGGTGCAGGTGCCACATTG	136	60
*CTSA*	NM_001311320.1	CGCTCGCTGAGAGGGTGATG	AGGCCGTGGTAGTAGGCGAA	124	60
*VDAC2*	XM_003451964.5	CCATCCACGGAGCTGCTGTC	ATGGAGCCGCCAAACTCTGC	172	60
*VDAC1*	XM_003451434.5	GGCTGGCTGGCTACCAGATG	CAGTCCAGGCCAGGTTGACG	189	60
*CTSD*	XM_003452585.5	CGAGCATCTCCGTGGATGGG	CAGGAGCAGCTCACCACCAG	131	60

## Data Availability

Data will be made available on request.
